# Reducing Inequities in Neonatal Mortality through Adequate Supply of Health Workers: Evidence from Newborn Health in Brazil

**DOI:** 10.1371/journal.pone.0074772

**Published:** 2013-09-20

**Authors:** Angelica Sousa, Mario R. Dal Poz, Cynthia Boschi-Pinto

**Affiliations:** 1 Department for Health Systems Policies and Workforce, World Health Organization, Geneva, Switzerland; 2 Institute of Social Medicine, University of the State of Rio de Janeiro, Rio de Janeiro, Brazil; 3 Department for Maternal, Newborn, Child and Adolescent Health, World Health Organization, Geneva, Switzerland; 4 Federal Fluminense University, Niterói, Rio de Janeiro, Brazil; Chancellor College, University of Malawi, Malawi

## Abstract

**Introduction:**

Progress towards the MDG targets on maternal and child mortality is hindered worldwide by large differentials between poor and rich populations. Using the case of Brazil, we investigate the extent to which policies and interventions seeking to increase the accessibility of health services among the poor have been effective in decreasing neonatal mortality.

**Methods:**

With a panel data set for the 4,267 Minimum Comparable Areas (MCA) in Brazil in 1991 and 2000, we use a fixed effect regression model to evaluate the effect of the provision of physicians, nurse professionals, nurse associates and community health workers on neonatal mortality for poor and non-poor areas. We additionally forecasted the neonatal mortality rate in 2005.

**Results:**

We find that the provision of health workers is particularly important for neonatal mortality in poor areas. Physicians and especially nurse professionals have been essential in decreasing neonatal mortality: an increase of one nurse professional per 1000 population is associated with a 3.8% reduction in neonatal mortality while an increase of one physician per 1000 population is associated with a 2.3% reduction in neonatal mortality. We also find that nurse associates are less important for neonatal mortality (estimated reduction effect of 1.2% ) and that community health workers are not important particularly among the poor. Differences in the provision of health workers explain a large proportion of neonatal mortality.

**Discussion:**

In this paper, we show new evidence to inform decision making on maternal and newborn health. Reductions in neonatal mortality in Brazil have been hampered by the unequal distribution of health workers between poor and non-poor areas. Thus, special attention to a more equitable health system is required to allocate the resources in order to improve the health of poor and ensure equitable access to health services to the entire population.

## Introduction

Despite appreciable progress, few of the 75 countries that account for more than 95% of all the maternal and child deaths globally are on track to attain the Millennium Development Goals (MDGs) on child and maternal mortality (MDGs 4 and 5) [1], [2]. One of the contributing factors to this slower than anticipated progress has been the lack of access to priority maternal and child health interventions among the poor [3].

In many countries, the levels of current staff are less than the minimum required to deliver health services [4] and in many others, health workers tend to be concentrated in better off areas limiting the access of health services to certain groups of the population [5], [6]. The quantity, distribution and quality of health workers which are accessible to the poor are key constrains for further gains towards the attainment of the MDGs by 2015.

Globally neonatal mortality (*deaths between 0–27 days*) represents 43% of overall under-five deaths and the proportion of under-five mortality attributed to neonatal mortality has increased [7]. In addition, recent figures show that progress towards reducing neonatal mortality rates has been slower in regions with high neonatal deaths [8]. Most of the neonatal mortality occur during the early neonatal period and is associated with inadequate care -lack of access to a functioning health facility or to qualified health professional- during and after pregnancy and child birth [9], [10]. It is therefore crucial that countries share experiences on policies and interventions that have been effective in decreasing neonatal mortality.

Brazil is the largest country with the largest economy in Latin America, but it also has large economic inequalities [11]; around 16 million people live in extreme poverty [12]. In Brazil, neonatal mortality accounts for 71% of infant mortality (this percentage has increased by 30% in the past 21 years) [7] and it is among the top 10 countries with the highest number of preterm births [13].

Over the last two decades Brazil has undergone a series of major health reforms with an emphasis on the universal provision of health services, particularly primary health care, combined with large scale decentralization of decision making. The 1988 constitution mandated universal access to health services and established a decentralized health system. The Single Health System (Sistema Unico de Saude –SUS) established in 1990, provides access to public facilities and pays for access to private facilities. While the system is designed to promote universal access there is a great deal of local autonomy in the level of provision, the pay of public sector workers and the reimbursements to the use of private facilities. Despite the progress made to decrease inequalities between the rich and the poor in several health indicators [14]–[16], major health inequalities remain [6], [17]–[27].

Sousa *et al.* 2010 & 2012 found that the poorest areas in Brazil suffer from worse neonatal and child health than richer areas and the poor- rich gaps have increased over time. The poorest states also experienced the highest inequalities in the distribution of physicians plus nurse professionals and at the same time have the highest shortage of skilled health workers [6], [25]–[27]. In this paper we assessed whether the differences in the supply of health workers and skill mix composition have measurable effects in the health of the population particularly on neonatal mortality. We focused on neonatal mortality as most of neonatal deaths could be averted with a functioning health system.

## Materials and Methods

To explore the relationship between the supply of health workers and neonatal mortality we constructed a panel data set with three different sources of information for two years, 1991 and 2000, on the 4,267 Minimum Comparable Areas (MCA) in Brazil. MCA is the unit of analysis rather than municipalities (the decentralized level of decision making), as it is the smallest geographical unit comparable between 1991 and 2000 population census. Data on neonatal mortality rate per 1000 live births for 1991 and 2000 are from Sousa et al. 2010a. Using sub-national data of neonatal mortality and child mortality rates from the three sub-national representative Demographic and Health Survey (DHS) conducted in Brazil; 1986, 1991 and 1996; the authors first investigated the relationship between neonatal and under five mortality per 1000 live births at sub-national level for the Southern and Northern Regions in Brazil (using a log-log regression model). They then extrapolated these relationships to predict neonatal mortality rates for the municipalities of the Southern and Northern Regions using publicly available data on under-five mortality per 1000 live births at municipality level for the 1991 and 2000 population censuses [27].

Data on health workers were extracted from the microdata of the population Census 1991 and 2000, using sample weights to produce four categories of health workers in the form of densities per 1000 population: physicians; nurse professionals; nurse associates (technicians, auxiliaries of nursing, nursing assistants, practical midwives and similar); and community health workers [28], [29].

We included as covariates in our analysis, the proportion of population living in urban areas, the population density per km^2^ and the proportion of population living below the poverty line defined as the proportion of population with a monthly family income per capita of less than 75$R, which is equivalent to half a minimum wage per capita in August 2000 (from the Institute of Applied Economic Research (IPEA) [30]. We then defined poor MCAs as those with more than 50% of population below the poverty line, and non-poor otherwise. Table 1 shows the national mean and standard deviation (SD) of the variables used in this analysis.

**Table 1:** Descriptive statistics.

	1991		2000	
	Mean	SD	Mean	SD
Neonatal mortality per 1000 live births	26.19	8.85	16.85	7.68
Physicians per 1000 population	0.303	0.601	0.320	0.587
Nurse professionals per 1000 population	0.054	0.227	0.112	0.293
Nurse associates per 1000 population	1.889	1.773	2.448	1.800
Community health workers per 1000 population	0.415	0.779	1.207	1.180
Population density per km2	0.122	0.422	0.104	0.313
% of population below the poverty line	57.10%	23.10%	45.40%	22.70%
% of urban population	53.70%	23.20%	61.80%	21.80%

Sources: Data from the population Census 1991 & 2000, the Institute of Applied Economic Research (IPEA) and Sousa A, et al. 2010 for neonatal mortality.

Note: For all variables, differences in the mean values between years are statistically significant except for the density of physicians per 1000 population.

### Methods

We used a fixed effect regression model with robust standard errors to evaluate the effect of the provision of health workers on neonatal mortality for poor and non-poor areas in Brazil, in 1991 and 2000. To evaluate the contributions of the different categories of health workers on neonatal mortality we included separately the densities of physicians, nurse professionals, nurse associates and community health workers. The statistical model used is:
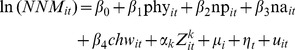
(1)where t = 1,2 and *i* = 1,.,nwhere *i* denotes the *i*-th MCA, *µ_i_* represents state fixed effects and *η_t_* time fixed effects. The ***Z^k^_it_*** vector of covariates is composed of a dummy for poor areas, the population density per km^2^ and a dummy for the proportion of the population living in urban areas. We additionally included a dummy variable to control for the fact that there are no health workers in some MCA. We did not include health expenditure as an additional explanatory variable in the model because it is highly correlated with the supply of health workers, as health workers account for approximately 70% of recurrent expenditure in most health systems [4]. We tried other variables such as the proportion of adult women with less than five years of education but it was not considered for the final analysis because of multicolinearity as it is highly correlated with the level of poverty. Moreover, it had less explanatory power than the variables finally included in the models. We also explored an alternative model, the random effect model, as an alternative specification, but we rejected it based on the Hausman test (rejected p<0.001) (which is the formal test for testing statistically significant differences between the coefficients in the time varying explanatory variables to discriminate between Fixed Effect and Random Effects models [31], [32]).

We additionally forecasted the neonatal mortality rate for poor and non-poor areas in 2005 using the predictive model of equation 1 and the density of health workers in 2005 extracted from Datasus [33], [34]. We then measured the trend of neonatal mortality rates between 1991 and 2005 for poor and non-poor areas. The analysis was performed using STATA 11 [35].

## Results

To estimate the effect between health worker availability (disaggregated by physicians; nurse professionals; nurse associates; and community health workers) and neonatal mortality rates, three models were estimated. Model 1, shows the results for the entire sample; Model 2, shows the results for poor MCAs and Model 3, for non-poor areas. Table 2 shows the coefficients, the signs and the level of significance of the parameters estimated by the fixed effect regression model with robust standard errors.

**Table 2:** Fixed-effect regression models of neonatal mortality rates for the MCA in all the sample and separated by poor and non-poor areas in Brazil, 1991–2000.

	All sample		Poor areas		Non-poor areas	
	Model 1		Model 2		Model 3	
Variables	Coef.	SE	Coef.	SE	Coef.	SE
Physicians per 1000 population	−0.0248***	(0.0054)	−0.0231*	(0.0108)	−0.0310***	(0.0057)
Nurse professionals per 1000 population	−0.0429***	(0.0100)	−0.0384**	(0.0141)	−0.0505***	(0.0114)
Nurse associates per 1000 population	−0.0115***	(0.0018)	−0.0117***	(0.0022)	−0.0083***	(0.0024)
Community health workers per 1000 population	0.0125***	(0.0029)	0.0005	(0.0031)	0.0130**	(0.0045)
Dummy for health workers availability	−0.0642***	(0.0125)	−0.0458**	(0.0145)	−0.0783***	(0.0206)
Dummy urban	−0.0056	(0.0059)	−0.0240***	(0.0063)	0.0041	(0.0116)
Dummy poor	0.2744***	(0.0083)				
Population density	0.0039	(0.0070)	0.0094	(0.0070)	−0.0036	(0.0210)
Dummy year	−0.4483***	(0.0057)	−0.3044***	(0.0068)	−0.5901***	(0.0084)
_cons	2.8878***	(0.0386)	2.9973***	(0.0396)	3.1268***	(0.0399)
N	8534		4471		4063	
r2	0.7825		0.5471		0.6765	
r2_a	0.7816		0.5437		0.6738	

Sources: Author’s calculation using data from the population Census 1991 & 2000, the Institute of Applied Economic Research (IPEA) and Sousa A, et al. 2010 for neonatal mortality.

Note: The models control for state fixed effects not presented in the table. Estimates were produced using robust standard errors to adjust for the presence of heteroscedasticity. We used the log of neonatal mortality as dependant variable. Statistical significance with a *p<0.05; **p<0.01; ***p<0.001. Poor refers to minimum comparable areas (MCA) with more than 50% of population below the poverty line, and non-poor otherwise. In all models, differences in the coefficients between categories of health workers are statically significant except for the densities of physicians and nurse professionals. Differences in the coefficients between poor and non-poor areas are also statistically significant. Other covariates such as the proportion of adult women (over age 15) with less than five years of education (average years) were also explored but not considered for the final analysis because of multicolinearity and for having less explanatory power than the variables finally included in the models.

In the first regression for all MCAs -Model 1-, we found that, after controlling for municipalities’ socioeconomic characteristics, the densities of physicians, nurse professionals and nurse associates have an inverse effect on neonatal mortality and these effects are highly significant. An increase of one physician per 1000 population is associated with a 2.5% reduction in neonatal mortality. Likewise, an increase of one nurse associates per 1000 population is associated with a 1.15% reduction in neonatal mortality. The highest impact is found for nurse professionals; where an increase of one nurse professional per 1000 population is associated with a 4.3% reduction in neonatal mortality. We also found an unexpected and statically significant association between the density of community health workers and neonatal mortality. A decrease in one community health worker per 1000 population is associated with a 1.25% reduction in neonatal mortality. Increasing the availability of all categories of health workers have a highly and significant effect in decreasing neonatal mortality of 6.4%.

When we separated the sample by poor and non-poor areas, in models 2 and 3, we also found that health workers have a significant and strong effect on neonatal mortality for poor and non-poor areas. In poor areas -Model 2-, it tends to be skilled health workers in the form of physicians and nurse professionals what matters for neonatal mortality. We found that the effect of physicians on decreasing neonatal mortality was of 2.3% while for nurse associates the effect was of 1.2%. Likewise, the highest effect on neonatal mortality was found for nurse professionals. An increase of one nurse professionals per 1000 population is associated with a 3.8% reduction in neonatal mortality. We also found that the presence of community health workers in poor areas is not associated with neonatal mortality.

For rich areas -Model 3-, we found that all categories of health workers have an inverse and significant effect on decreasing neonatal mortality except for community health workers which have again an unexpected and statistically significant association with neonatal mortality.

For the three models, the time variable has an inverse and significant relationship with neonatal mortality, which implies that mortality rates decrease over time as an effect of the technological change. We found that the change in technology has contributed to a 30% reduction in NMR in poor areas and to a 59% in non-poor areas. The socioeconomic determinants of neonatal mortality in the regressions are urbanization, population density and the level of poverty. We found that population density is not statistically significant in any of the three models. For all MCAs -Model 1-, poverty is significantly associated with neonatal mortality. In the poor areas -Model 2-, urbanization is inversely associated with neonatal mortality, while for non-poor areas -Model 3- it is not statistically significant.

We found that the total absolute reduction in neonatal mortality for poor areas during the period analysed was of 7.6 deaths per 1000 live births (from 27.9 per 1000 live births in 1991 to 20.3 in 2000). In non-poor areas, the total reduction in neonatal mortality in the same time period was of 9.5 deaths per 1000 live births (from 20.9 per 1000 live births in 1991 to 11.4 in 2000). For policy implications, we estimated the contributions of skilled health workers -physicians and nurse professionals- and unskilled health workers -community health workers and nurse associates- to the reduction of neonatal mortality in poor and non-poor areas (see Table 3). In poor areas the increase of health workers availability has contributed to a 27% reduction of neonatal mortality while in richer areas their contribution has been of 17%. In addition, the marginal effect of raising the number of skilled health workers in poor areas to the average level found in rich areas would decrease the proportion of neonatal mortality by about 6%.

**Table 3:** Effect of skilled and unskilled health workers availability on neonatal mortality in poor and rich areas.

	Poor areas	Non-poor areas
Explained reduction by skilled health workers	−6.15%	−8.15%
Explained reduction by unskilled health workers	−1.17%	0.47%
Total explained reduction	−7.32%	−7.68%
Total reduction in neonatal mortality between 1991–2000	7.6	9.5
Percentage explained reduction by skilled health workers	22.80%	18.11%
Percentage explained reduction by all health workers	27.10%	17.10%

Sources: Author’s calculation using the output from Table 2.

Note: Skilled health workers refers to physicians & nurse professional and unskilled health workers to nurse associate & community health workers. Poor refers to minimum comparable areas (MCA) with more than 50% of population below the poverty line, and non-poor otherwise. The explained reduction by skilled health workers for poor and non-poor areas is the sum of the marginal effects estimated in Table 3 for physicians and nurse professionals. Similarly, the explained reduction by unskilled health workers is the sum of the marginal effects for nurse associate and community health worker.

We forecasted the neonatal mortality rate in 2005 using the coefficients from equation 1 and data on the density of health workers per 1000 population from 2005. We found that the projected neonatal mortality rate in 2005 at national level is of 13.1 per 1000 live births, which is similar to the estimated neonatal mortality rate at national level from Barros et al. 2010 of 13.6 in 2007 [20] and to the UN estimates of around 14 per 1000 live births in 2005 [7]. This consistency implies that our projected neonatal mortality rate can be disaggregated for poor and non-poor areas.

**Figure 1:**
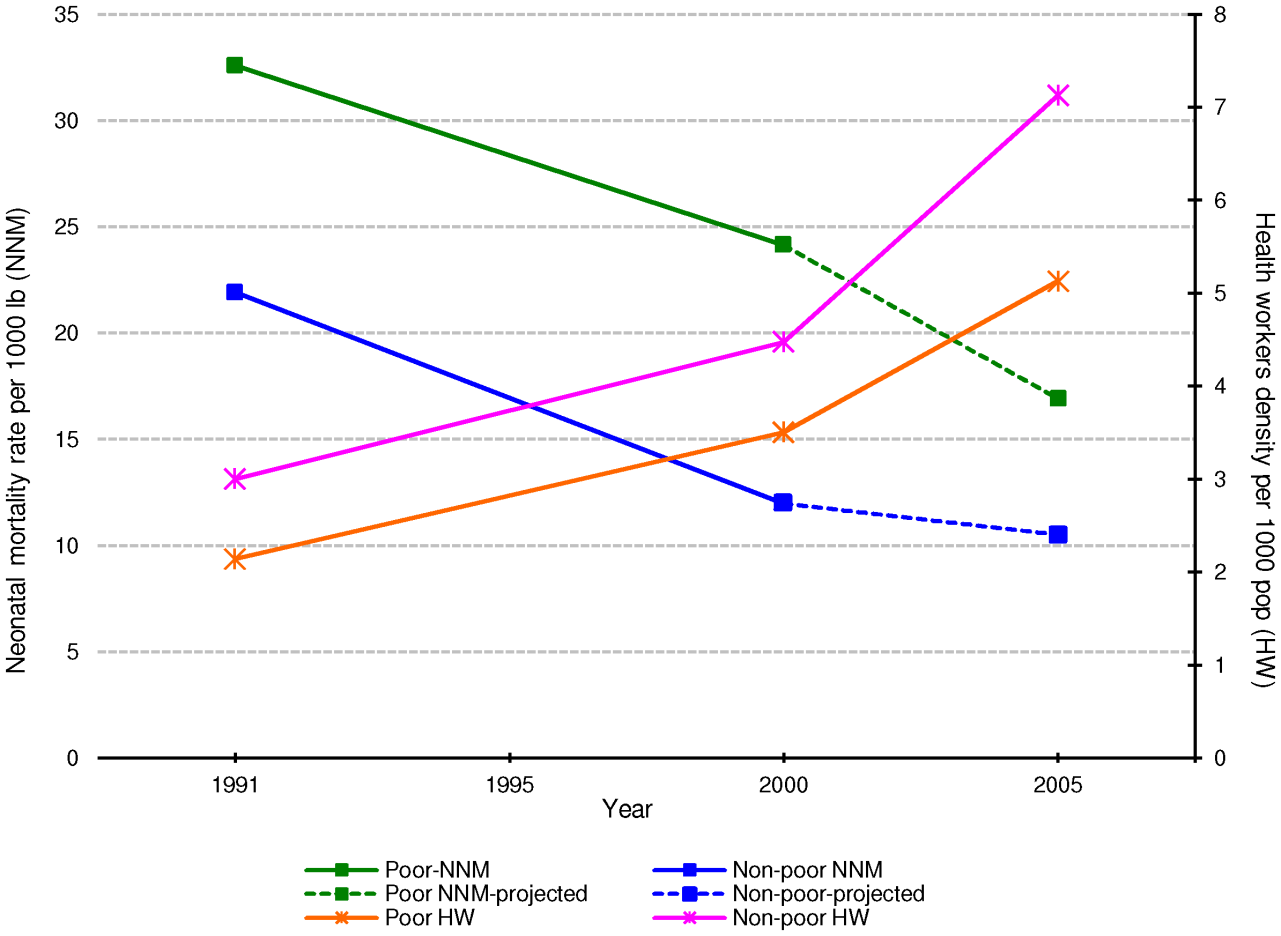
Trends of the neonatal mortality rate per 1000–2005. Sources: Author’s calculation using data from the population Census 1991 & 2000, the Institute of Applied Economic Research (IPEA), DATASUS 2005, Sousa A, et al. 2010 for neonatal mortality 1991 & 2000 and projected estimates of neonatal mortality rate 2005 from output table 2. Note: X axis = year. Y axis left = neonatal mortality rate per 1000 lb. Y axis right = health workers density per 1000 pop. Green square = neonatal mortality rate for poor areas. Blue diamond = neonatal mortality rate for non-poor areas. Pink cross = health workers density for non- poor areas. Orange cross = health workers density for poor areas.

Figure 1 shows the trends of the neonatal mortality rate per 1000 live births and health workers density per 1000 population for poor and non-poor areas between 1991 and 2005. In general, we found that between 1991 and 2005 there has been a constant decline in neonatal mortality in both poor and non-poor areas and, at the same time, there has been a sharp growth on the availability of health workers per 1000 population. However we found great inequalities; poorer areas have higher neonatal mortality rate and lower density of health workers than richer areas and this problem has not changed over time. The projected neonatal mortality rate in 2005 in poor areas is of 17 per 1000 live births while for non-poor areas the projected neonatal mortality rate is of 10.5 per 1000 live births.

## Discussion

Our analysis suggests that larger gains in neonatal mortality have been hindered by the large differentials in the availability of health workers between the poor and non-poor areas in Brazil. Differences in the provision of skilled health workers explain a large proportion of neonatal mortality in the poorest areas. Thus, policies and interventions seeking to increase the availability of qualified health workers would have a dramatic impact in decreasing neonatal mortality among the poor.

We demonstrated that qualified health workers are essential in decreasing neonatal mortality particularly among the poor. Although nurse professionals have the highest impact on decreasing neonatal mortality, they remain the category of health workers the less deployed across the country. We also found that nurse associates are less important for neonatal mortality and that community health workers do not have an effect particularly among the poor. Since these categories of health workers have mixed skills, training and education, our findings imply that their training on neonatal care should be harmonized across different areas of the country and further strengthened to have an impact on neonatal mortality as demonstrated in other areas of the world. For example, the positive impact of home visits by community health workers on neonatal mortality has been clearly demonstrated by several randomized and non-randomized controlled trials. A meta-analysis of 8 studies involving more than 65,000 babies has showed a significant 63% reduction in neonatal mortality among those newborn babies receiving home visits by a community health worker compared to those who did not receive the intervention [36]–[39].

Other options to increase the accessibility of health services in low resource settings and decrease the rate of neonatal mortality could include: 1) strengthening the education of mid-level health workers; 2) more efficient utilization of health worker time by increasing the productivity of the current health workforce [26]; 3) implementing strategies to retain health workers in underserved areas; and 4) changing the skill mix of health workers by using health workers with less training to carry out a variety of healthcare tasks if they receive appropriate training [40]–[42].

The large socioeconomic and health inequalities that remain in Brazil, together with the percentage increase of neonatal mortality on child mortality, raise major concerns for further improvements on child mortality [16], [20]. Thus this analysis makes a significant contribution for Brazil and the experience presented in this paper is relevant for other developing countries undergoing similar challenges in decreasing neonatal mortality, as it quantifies the effect of implementing health sector policies to decrease the poor/non-poor gap in neonatal mortality across sub-national areas.

There are some limitations of this analysis that merit consideration. Due to data limitations we have used the same approximation as Farahani et.al. 2010 [43], which considered the number of physicians as a proxy for health system resources in general, and assumed that other inputs usually vary proportionately with the number of health workers, so that our results represent the effect of health inputs as a whole and not just the impact of the number of the different categories of health workers. We have restricted this analysis to the different categories of health workers supply, since panel data on other health resources are lacking for all MCA. Since we account for state fixed effects in our analysis, we have controlled for any confounding variables that are fixed in a state over time. Even though municipality is the level of decision making after the decentralization reform, we did not use municipality as the unit of analysis as it is not comparable over time, the reason being that more than one thousand municipalities were created between 1991 and 2000. We therefore used data of the 4,267 MCAs to eliminate the problem of comparability in the unit of analysis. We used estimates of the neonatal mortality for the MCA produced in Sousa et.al. 2010 [27] as the national civil registration data are not an accurate source of information on neonatal deaths, particularly for the poorest regions in Brazil [44]–[47]. We were also unable to adjust for health workers quality: training, re- training, experience (as measured by length of service) and individual skills for all categories of health workers are key elements to ensure quality services. Although most categories of health workers used in our analysis were clearly defined, the umbrella term “community health worker” embraces a variety of community health aides, which are likely to range from untrained volunteers from the community to well trained professionals. This implies that our results may have been affected by the heterogeneity in the definition of community health workers as well as the impossibility to assess the quality of training and their individual skills. These limitations mean that our results should be interpreted with caution. Further studies, in Brazil as well as in other countries of the world, should be conducted using more recent data to verify the findings of this study.

Despite the limitation of the data, this study has highlighted critical issues in terms of the quantity, distribution and skill mix of health workers which are accessible to the poor. Despite the commitment to universal access to health services in Brazil and expansion of health services among the poor, Sousa et al. 2012 found that in practice poor areas have fewer health workers than rich areas and in addition have the highest inequalities in the distribution of skilled health workers. The majority of the staff in poor areas are community health workers and poor communities have very few physicians and nurse professionals [6]. In this paper we demonstrated that this lack of provision, relative to richer municipalities, translates into large gaps in neonatal mortality. The evidence in this paper suggests that addressing the imbalances in the distribution of health workers between poor and non-poor areas would be key to improve child health in poor areas. Thus, special attention to a more equitable health system is required to allocate the resources in order to improve the health of poor and ensure equitable access to health services to the entire population to attain universal health coverage.
